# NFE2L3 as a Novel Biomarker Associated With IL-2/STAT5/NLRP3 Signaling Pathway in Malignant Pleural Mesothelioma and Other Cancers

**DOI:** 10.3389/fgene.2022.805256

**Published:** 2022-05-18

**Authors:** Zhen Wang, Han Yang, Bin Luo, Pengfei Duan, Peng Lin

**Affiliations:** Department of Thoracic Oncology, State Key Laboratory of Oncology in South China, Collaborative Innovation Center for Cancer Medicine, Sun Yat-sen University Cancer Center, Guangzhou, China

**Keywords:** NFE2L3, IL-2, Stat5, NLRP3, t-helper 2 cell, malignant pleural mesothelioma

## Abstract

**Background:** Malignant pleural mesothelioma (MPM) is a malignant tumor originating from pleural mesothelial cells and has a high mortality rate worldwide. With the advent of immunotherapy in MPM treatment, there is an urgent need to elucidate the immune-related mechanisms in this caner.

**Methods:** Single-sample gene set enrichment analysis (ssGSEA) was used to score the immunocytes infiltration of data from different database sources. Identification of immunocyte-related genes was performed with weighted gene co-expression network analysis (WGCNA), differentially expressed genes (DEGs) analysis, and correlation analysis. Pan-caner analysis was performed using “DiffExp” and “Correlation” modules in TIMER.

**Results:** T-helper 2 (Th2) cell was found to be a poor prognostic factor for patients with MPM. Then a transcription factor, NFE2L3, was identified as a biomarker that showed a strong positive correlation with Th2 cell infiltration, and was highly expressed in MPM tissues and was related to the poor prognosis of these patients. At the same time, multiple NFE2L3 methylation sites were negatively correlated with Th2 cell infiltration, and patients with a high degree of methylation enjoy a better prognosis. Pan-caner analysis indicated that NFE2L3 might promote the differentiation of Th2 cells through the IL-2/STAT5/NLRP3 signaling pathway in MPM and many other cancers.

**Conclusion:** We believe that NFE2L3 can serve as a potential biomarker related to the diagnosis and prognosis of patients with MPM, and speculate that NFE2L3 could promote Th2 cell differentiation *via* IL-2/STAT5/NLRP3 signaling pathway in MPM and many other cancers.

## Introduction

Malignant pleural mesothelioma (MPM) originates from pleural mesothelial cells and is a relatively rare type of cancer, which accounts for 0.3% of all cancer cases (Fernandez-Cuesta et al., 2021). Due to its aggressiveness and difficulty in early diagnosis, MPM is difficult to be cured. Because of the limited role of surgery in the treatment of MPM, the chemotherapy regimen of pemetrexed combined with platinum has occupied a dominant position in the treatment of MPM for a long time (de Gooijer et al., 2018). However, survival benefit from chemotherapy is limited, and the 5-years survival rate of patients with MPM is still less than 10% (Kindler et al., 2018).

In recent years, immune checkpoint inhibitors (ICIs) have been proven to improve the prognosis of various solid tumors, and their anti-tumor effects in MPM have gradually become clear (Lievense et al., 2017). A phase III clinical trial (Checkmate 743) has proved for the first time that compared with chemotherapy, the first-line treatment of nivolumab combined with ipilimumab can provide significant improvements in overall survival (OS) for patients with advanced MPM (Baas et al., 2021). However, considering the complexity of the tumor immune microenvironment, we still need to explore more immune-related mechanisms and targets to increase our understanding of MPM and treatment methods for this disease.

As an essential part of the human immune system, CD4^+^ T cells play a pivotal role in adaptive immune responses. Under the activation of external cytokines, naive CD4^+^ T cells can differentiate into multiple T helper cells, including Th1, Th2, Th17 (Zhu et al., 2010). Among them, Th2 cells have been confirmed to have immunosuppressive effects in many tumors (Kusuda et al., 2005; Nevala et al., 2009; De Monte et al., 2011). In normal tissues, T helper lymphocyte subsets are in equilibrium. However, tumor cells can secrete a variety of cytokines, putting the body in a state where Th2 cells dominate, leading to immune escape and tumor progression. Therefore, in this study, we focused on the effect of Th2 cells in patients with malignant pleural mesothelioma and explored a novel regulatory network of Th2 cell differentiation using a series of bioinformatics analysis methods.

## Materials and Methods

### Analysis Overview

**FIGURE 1:**
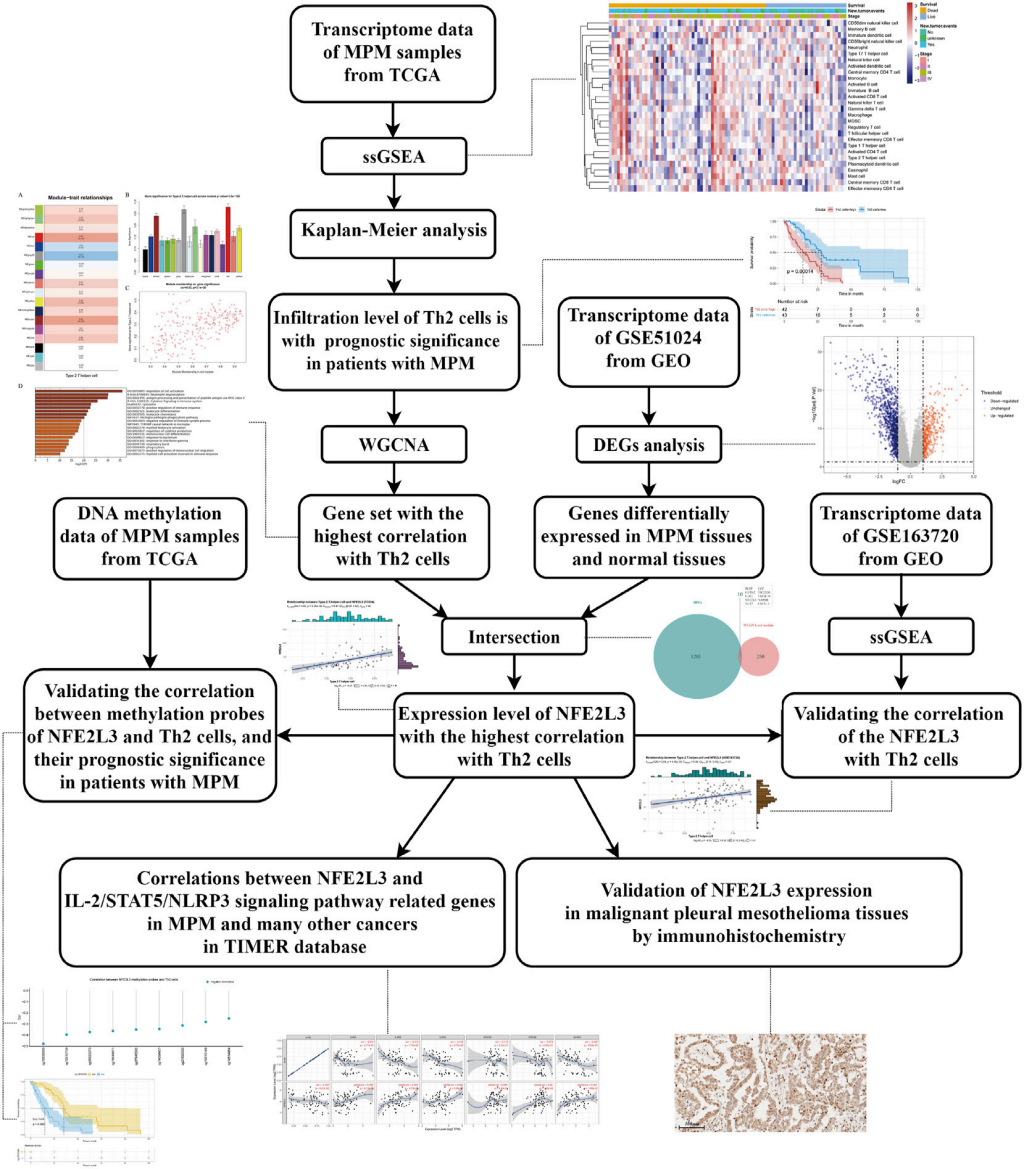
The detailed workflow of this study. MPM, malignant pleural mesothelioma; TCGA, The Cancer Genome Atlas; ssGSEA, single-sample gene set enrichment analysis; Th2 cells, type 2 T helper cells; WGCNA, weighted gene co-expression network analysis; GEO, Gene Expression Omnibus; DEGs, differentially expressed genes.

In this study, utilizing transcriptomes downloaded from TCGA and GEO, we firstly identified that Th2 cell was associated with prognosis of patients with MPM, and NFE2L3, a transcription factor (TF), was associated with infiltration of this immunocyte. Then, using DNA methylation data from TCGA, we analyzed the correlation of NFE2L3’s methylation site with the infiltration of Th2 cell and the prognosis of patients with MPM. Meanwhile, using tissue sections from patients with MPM, we verified the expression of NFE2L3 in normal pleural tissues and tumor tissues. Finally, with the help of TIMER database, we analyzed the correlation between NFE2L3 and Th2 cell regulatory pathway-related genes in mesothelioma (MESO) and many other cancers. The detailed research process is shown in the flow chart (Figure 1).

### Date Source

All data used in this study are from public databases, including Gene Expression Omnibus (GEO) and The Cancer Genome Atlas (TCGA) (Barrett et al., 2013; Cancer Genome Atlas Research et al., 2013). The clinical data, transcriptome data in FPKM format and DNA methylation data of malignant pleural mesothelioma (MPM) in TCGA were downloaded from the National Cancer Institute’s (NCI’s) Genomic Data Commons (GDC) (https://portal.gdc.cancer.gov/) (Zhang et al., 2021). Search with “mesothelioma” as a keyword, we found and downloaded gene expression profiles of GSE51024 and GSE163720 from GEO database (http://www.ncbi.nlm.nih.gov/geo/). GSE51024 is composed of 55 MPM tissues and 41 normal paired lung parenchyma tissues. And GSE163720 contains 131 tumor samples from patients with MPM.

### Single-Sample Gene Set Enrichment Analysis

Immunocyte-related gene sets were got from The Cancer Immunome database (TCIA) (Charoentong et al., 2017). Through the expression value of 782 immune-related genes, we scored infiltration levels of 28 types of immunocytes. Immunocytes infiltration levels of samples were quantified by the ssGSEA algorithm in R package GSVA (Hanzelmann et al., 2013). Through Kaplan-Meier survival analysis, we found out the target immunocyte associated with the prognosis of patients with MPM.

### Weighted Gene Co-Expression Network Analysis

The transcriptome data of samples from TCGA were further analyzed utilizing WGCNA to find a gene set highly correlated with infiltration level of the target immunocyte. As an algorithm for transcriptome analysis, WGCNA can identify genes with highly correlated expression patterns, and calculate the correlation between the gene set and clinicopathological traits of samples (Langfelder and Horvath, 2008).

R package “WGCNA” was used to complete the calculation process in this step. First, the 5,000 genes with the highest average expression were selected for the subsequent analysis. Then, the optimal soft-thresholding power for network construction was calculated, and module eigengenes (MEs) containing a series of co-expressed genes were constructed using a dynamic tree-cutting algorithm. Finally, the co-expressed gene set with the strongest correlation with Th2 cell infiltration can be found by analyzing the correlation between MEs and clinicopathological traits.

### Functional Enrichment Analysis

Metascape, a meta-analysis website, was used for functional enrichment analysis of the co-expressed gene set (Zhou et al., 2019). By using the “Express Analysis” module in Metascape, the enriched biological processes and pathways of the selected co-expressed gene set were obtained.

### Differentially Expressed Genes and Correlation Analysis

GSE51024 is composed of 96 samples, including 55 MPM tissues and 41 normal paired lung parenchyma tissues. Using GSE51024 for DEGs analysis, we can screen out genes that are significantly up-regulated or down-regulated in MPM tissues compared to normal lung parenchyma tissues. DEGs were analyzed using “limma” package in R, with an adjusted *p* value < 0.05 and |logFC| >1 (Ritchie et al., 2015). Mann-Whitney test was performed to calculate expression differences between tumor and normal tissues (Perme and Manevski, 2019).

The intersection of DEGs and selected MEs was used to further analyze the correlation with infiltration level of the target immunocyte. Transcriptome data and methylation data from TCGA was used for correlation analysis, and GSE163720 from GEO was used for verification. Spearman’s correlation test was performed to screen the gene with the highest correlation with infiltration level of the target immunocyte (Bishara and Hittner, 2012). And the correlation coefficient greater than 0.3 is considered vital (Funder and Ozer, 2019). Then, Kaplan-Meier survival analysis was used to explore the relationship between transcriptome data and methylation data of the screened gene and prognosis of patients with MPM.

### Immunohistochemistry Staining

MPM and normal pleural tissues were obtained from patients who undergone surgery in Sun Yat-Sen University Cancer Center. All specimens were diagnosed as MPM by pathologist in Sun Yat-Sen University Cancer Center.

Immunohistochemistry (IHC) staining was used to examine NFE2L3 expression in MPM tissues and paired normal pleural tissues. All paraffin-embedded specimens were cut into 5-μm sections and placed on glass slides, then baked at 60°C for 1 h. Firstly, dewaxing all specimens with xylene and rehydrating with ethanol, then immerse the specimens in sodium citrate-EDTA buffer, and using microwave heating for antigen retrieval. Secondly, using 3% hydrogen peroxide to inactivate endogenous peroxidase, and then blocking non-specific binding with 10% goat serum. Thirdly, incubating the slides with anti-NFE2L3 rabbit polyclonal antibody (1:200; NBP2-30870; Novus Biologicals) overnight at 4°C, after washing with PBS for 4 times, then adding secondary antibody polymer horseradish peroxidase to sections. Finally, the slices wer stained with DAB (3ʹ-diaminobenzidine) and hematoxylin sequentially, dehydrated with gradient ethanol and mounted with neutral resin.

### Pan-Cancer Analysis

TIMER (Tumor IMmune Estimation Resource) is a website that can provide a comprehensive analysis of transcriptome data from TCGA (Li et al., 2020). First, we study the differential expression of the screened gene between tumor and normal tissues across all TCGA samples using “DiffExp” module in TIMER. Then, using “Correlation” module, we performed correlation analysis of two related genes not only in MESO but also in many other cancer types. In this step, correlation analysis was adjusted by tumor purity.

## Results

### Patients With High Th2 Cell Infiltration Suffer a Poor Prognosis

**FIGURE 2:**
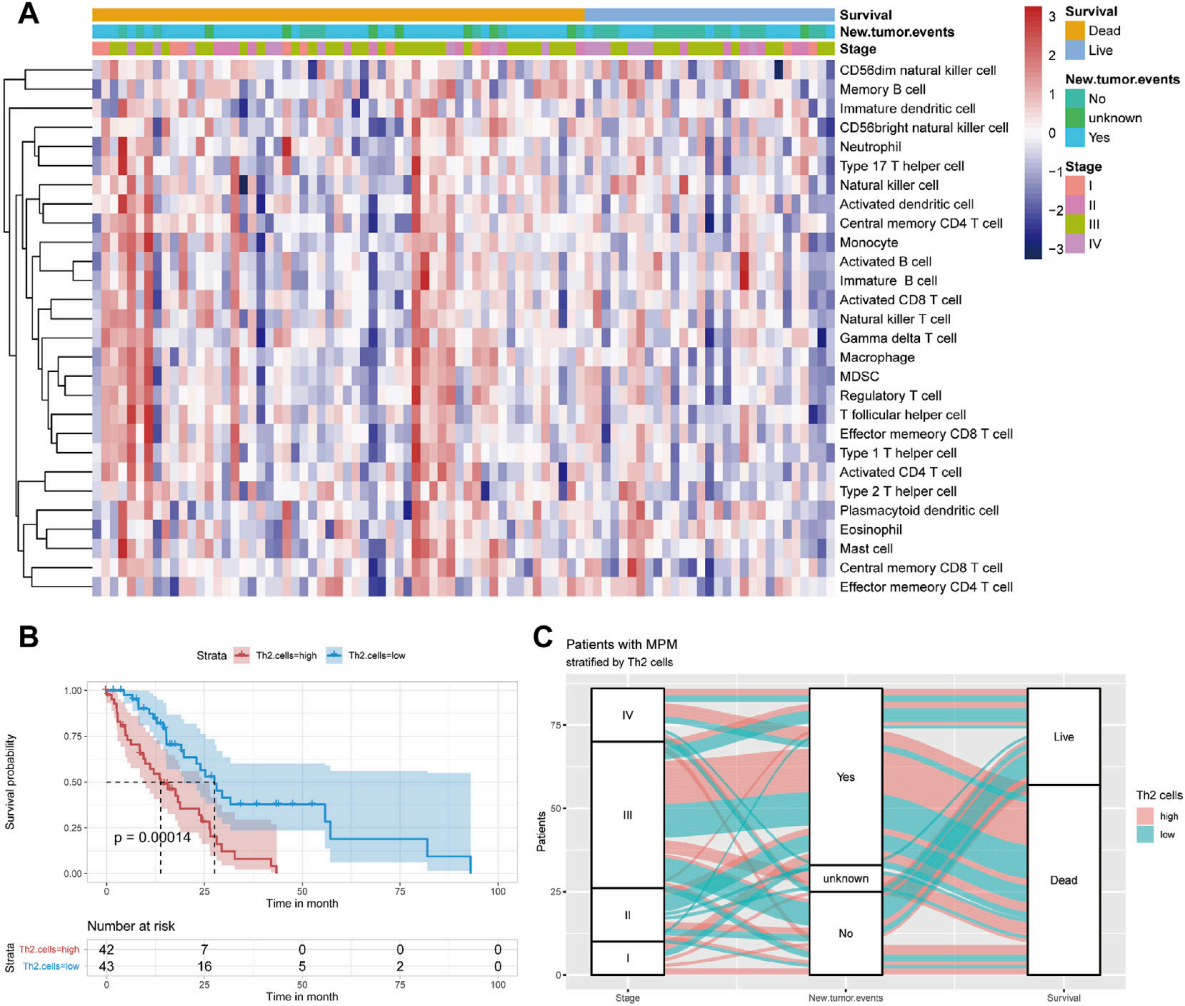
**(A)** Immunocyte infiltration levels of 85 samples from TCGA. **(B)** Kaplan–Meier curve of patients from TCGA when using the median of Th2 cell infiltration levels as the cut-off value **(C)** Sankey plot depicting the relationship across the tumor stage, new tumor event and survival status of patients stratified by median value of Th2 cell infiltration levels.

The heat map of the infiltration level of 28 immunocytes is shown in Figure 2A. Through Kaplan-Meier survival analysis, it can be seen that patients with high Th2 cell infiltration suffer a significantly poor prognosis (Figure 2B, *p* = 0.00014). A Sankey plot presents the correlation between stage, recurrence and survival status of patients stratified by Th2 cell infiltration level (Figure 2C).

### Identification of Co-Expressed Genes Associated With Th2 Cell

**FIGURE 3:**
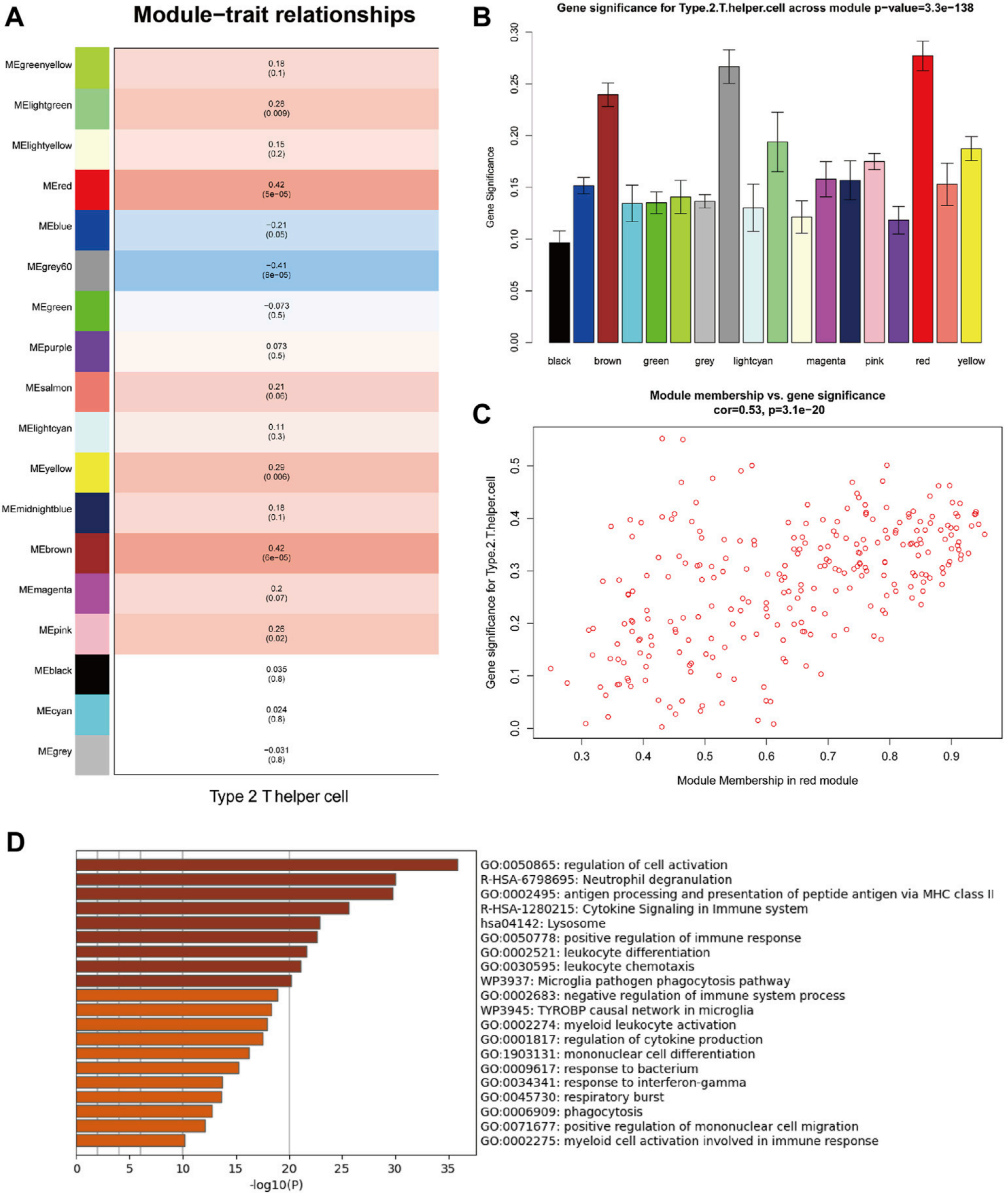
Weighted gene co-expression network analysis (WGCNA) of MPM samples from TCGA. **(A)** Correlations between module eigengenes (MEs) and Th2 cell infiltration levels. **(B)** Gene significance (GS) for Th2 cell infiltration levels across all modules. **(C)** Gene significance (GS) of the genes contained in the red module versus module membership (Th2 cell infiltration levels). **(D)** Functional enrichment analysis of genes contained in the red module (Functional enrichment terms colored by *p*-values).

WGCNA can identify co-expressed genes that are highly correlated with Th2 cell infiltration. With the help of a dynamic tree-cutting algorithm, the 5,000 genes with the highest average expression were divided into 18 module eigengenes (MEs). Then Pearson’s correlation coefficient was used to calculate the correlation between the MEs and clinicopathological traits. Figures 3A,B shows that the red module exhibits the highest correlation coefficient with Th2 cells (*Cor* = 0.42, *P* = 5e-05). The correlation across each gene in the red module and Th2 cells is plotted in Figure 3C (*Cor* = 0.53, *p* = 3.1e-20).

Functional enrichment analysis revealed that genes contained in the red module are mainly involved in various functions of the immune system (Figure 3D), including regulation of cell activation, neutrophil degranulation, antigen processing and presentation of peptide antigen via MHC class II, cytokine signaling in immune system, lysosome, positive regulation of immune response, leukocyte differentiation, leukocyte chemotaxis, microglia pathogen phagocytosis pathway and negative regulation of immune system process.

### Identification of Th2 Cell Infiltration-Related Genes

**FIGURE 4:**
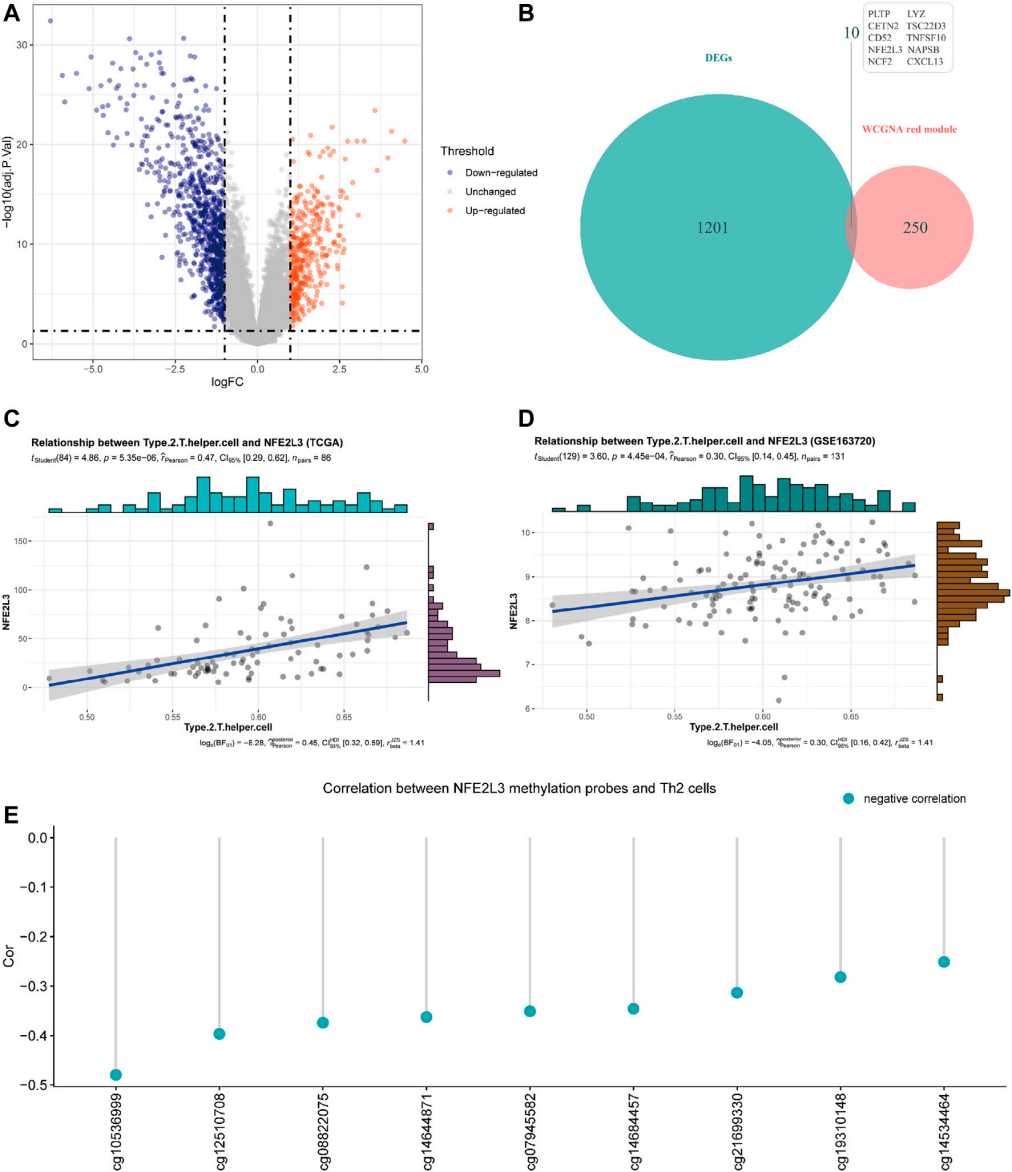
**(A)** Volcano plot of differentially expressed genes (DEGs) between tumor tissues and paired normal tissues of samples from GSE51024 (adjusted *p* value < 0.05 and |logFC| >1). **(B)** Venn plot of the intersection of DEGs from GSE51024 and genes contained in red module from WGCNA **(C)** Correlation between NFE2L3 expression and Th2 cell infiltration levels of samples from TCGA. **(D)** Correlation between NFE2L3 expression and Th2 cell infiltration levels of samples from GSE163720 **(E)** Correlations between DNA methylation levels of NFE2L3 related sites and Th2 cell infiltration levels of samples from TCGA.

Differentially expressed genes (DEGs) analysis was performed to identify up-regulated and down-regulated genes between MPM tissues and normal lung parenchyma tissues. Through DEGs analysis of the transcriptome data of GSE51024, 1,211 differentially expressed genes were screened, including 364 genes up-regulated in MPM tissues and 847 down-regulated genes. The expression profile of DEGs is shown in a volcano map (Figure 4A, adjusted *p* value <0.05 and |logFC| >1).

The red module obtained by WGCNA of data from TCGA contains 260 genes, of which 10 genes are differentially expressed in MPM tissues (Figure 4B). Through correlation analysis with Th2 cell infiltration level of samples from TCGA, NFE2L3 was identified that its expression level has the highest correlation with Th2 cell infiltration level (Figure 4C, *Cor* = 0.47). Then we utilized the transcriptome data of GSE163720 to validate and confirmed the strong correlation between NFE2L3 and Th2 cells (Figure 4D, *Cor* = 0.30).

**FIGURE 5:**
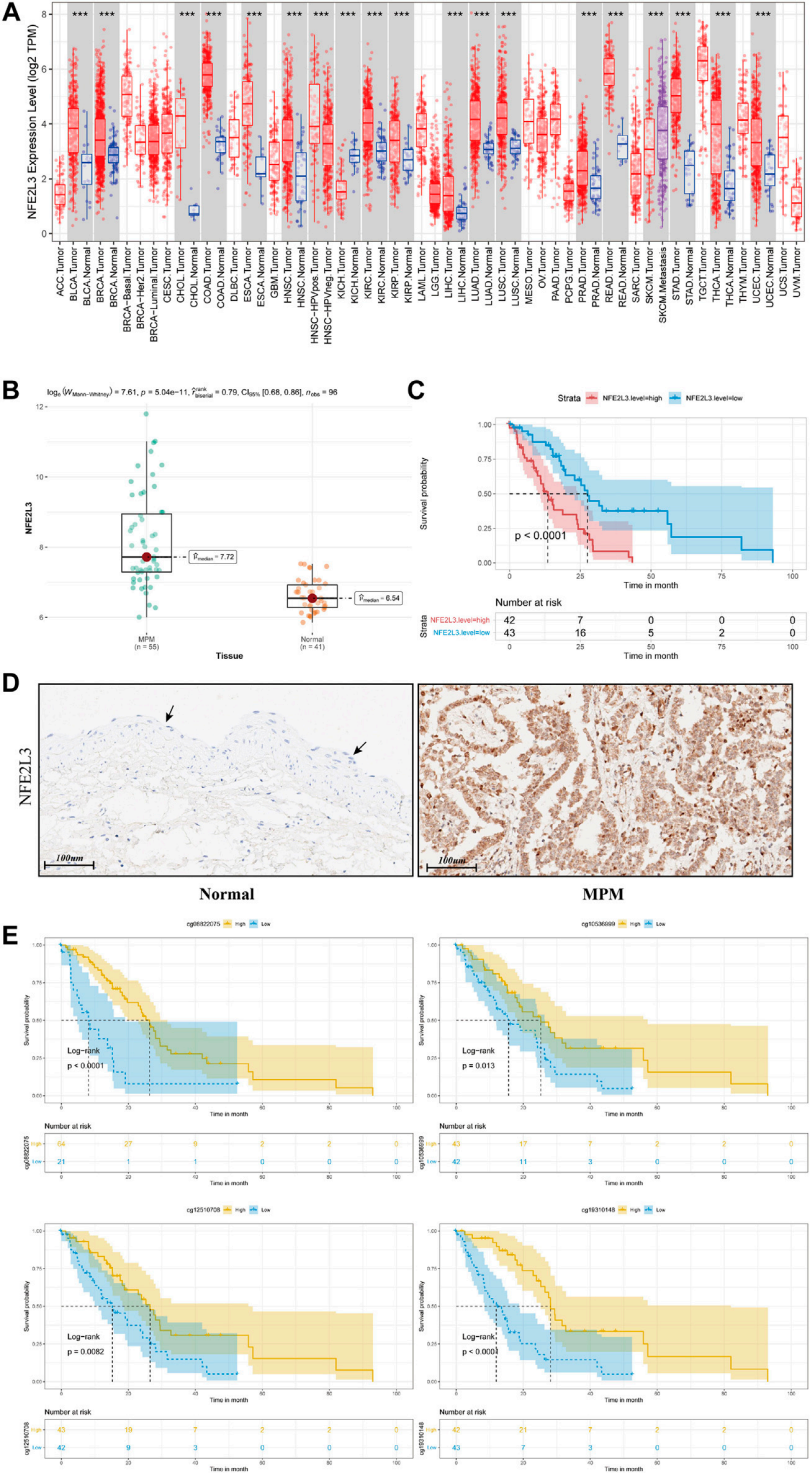
**(A)** Pan-cancer analysis of NFE2L3 expression of all samples in TCGA. **(B)** Box plot of NFE2L3 expression in tumor tissues and paired normal tissues of samples from GSE51024 **(C)** Kaplan–Meier curve of patients from TCGA when using the median of NFE2L3 expression as the cut-off value. **(D)** Immunohistochemical staining of NFE2L3 in normal pleural epithelial tissue (black arrow) and malignant pleural mesothelioma tumor tissue **(E)** Kaplan–Meier curves depicting the relationships between DNA methylation levels of NFE2L3 related sites and prognosis of patients with MPM.

By comparing MPM tissues and normal lung parenchyma tissues of GSE51024, NFE2L3 is highly expressed in MPM tissues (Figure 5B, *p* = 5.04e-11). And using tissue sections for immunohistochemical staining, we can observe that NFE2L3 is mainly expressed in tumor cell nuclei, but not in normal pleural tissues (Figure 5D). It is also confirmed by analyzing the transcriptome data of TCGA that patients with higher NFE2L3 expression suffer a worse prognosis (Figure 5C, *p* < 0.0001).

In TCGA, DNA methylation levels of MPM tissues were determined with the Illumina Infinium Methylation 450 K array. After matching sample names, 9 of the 19 methylation sites of NFE2L3 were found to be correlated with the infiltration of Th2 cells, and all of them were negatively correlated (Figure 4E). Table 1 details the basic information of NFE2L3 methylation sites and their correlations with Th2 cell infiltration level. Kaplan-Meier survival analysis also identified that the methylation levels of cg08822075, cg10536999, cg12510708, and cg19310148, were associated with the prognosis of patients, and patients with higher methylation levels had better prognosis (Figure 5D).

**TABLE 1:** The basic information of DNA methylation sites of NFE2L3 and the correlations between their methylation levels and Th2 cell infiltration. Chrom, chromosome; ChromStart, starting position in the chromosome; ChromEnd, end position in the chromosome.

	Gene	Chrom	ChromStart	ChromEnd	Correlation Between NFE2L3 Methylation Probes and Th2 Cells	*p*-Value
					Cor	
cg03781084	NFE2L3	chr7	26,152,106	26,152,107	−0.110	3.15E-01
cg03886242	NFE2L3	chr7	26,152,412	26,152,413	−0.019	8.63E-01
cg04995722	NFE2L3	chr7	26,152,414	26,152,415	0.009	9.36E-01
cg07876897	NFE2L3	chr7	26,152,076	26,152,077	−0.103	3.45E-01
cg07945582	NFE2L3	chr7	26,166,959	26,166,960	−0.351	9.19E-04^*^
cg07986525	NFE2L3	chr7	26,152,579	26,152,580	−0.166	1.26E-01
cg08822075	NFE2L3	chr7	26,153,987	26,153,988	−0.374	3.86E-04^*^
cg10536999	NFE2L3	chr7	26,153,489	26,153,490	−0.480	2.96E-06^*^
cg12510708	NFE2L3	chr7	26,154,185	26,154,186	−0.397	1.55E-04^*^
cg13118545	NFE2L3	chr7	26,151,979	26,151,980	−0.110	3.15E-01
cg13855897	NFE2L3	chr7	26,186,769	26,186,770	−0.002	9.82E-01
cg14534464	NFE2L3	chr7	26,152,013	26,152,014	−0.251	1.97E-02^*^
cg14644871	NFE2L3	chr7	26,153,136	26,153,137	−0.363	5.96E-04^*^
cg14684457	NFE2L3	chr7	26,153,346	26,153,347	−0.346	1.11E-03^*^
cg16882373	NFE2L3	chr7	26,151,838	26,151,839	−0.123	2.58E-01
cg18844118	NFE2L3	chr7	26,151,869	26,151,870	−0.043	6.95E-01
cg19310,148	NFE2L3	chr7	26,156,654	26,156,655	−0.282	8.54E-03^*^
cg21699330	NFE2L3	chr7	26,153,412	26,153,413	−0.314	3.29E-03^*^
cg24424745	NFE2L3	chr7	26,160,252	26,160,253	−0.051	6.39E-01

### NFE2L3 Could Promote Th2 Cell Differentiation via IL-2/STAT5/NLRP3 Signaling Pathway

Through pan-cancer analysis of samples in TCGA, it can be seen that compared with normal tissues, NFE2L3 is highly expressed in almost all kinds of tumor tissues (Figure 5A).

According to previous research, NLRP3 is a transcriptional regulator of Th2 cell differentiation, and signal transducer IL-2R and STAT5 triggers its expression (Bruchard et al., 2015). Therefore, we further studied the correlation between NFE2L3 and the expression of related genes involved in this pathway.

**FIGURE 6:**
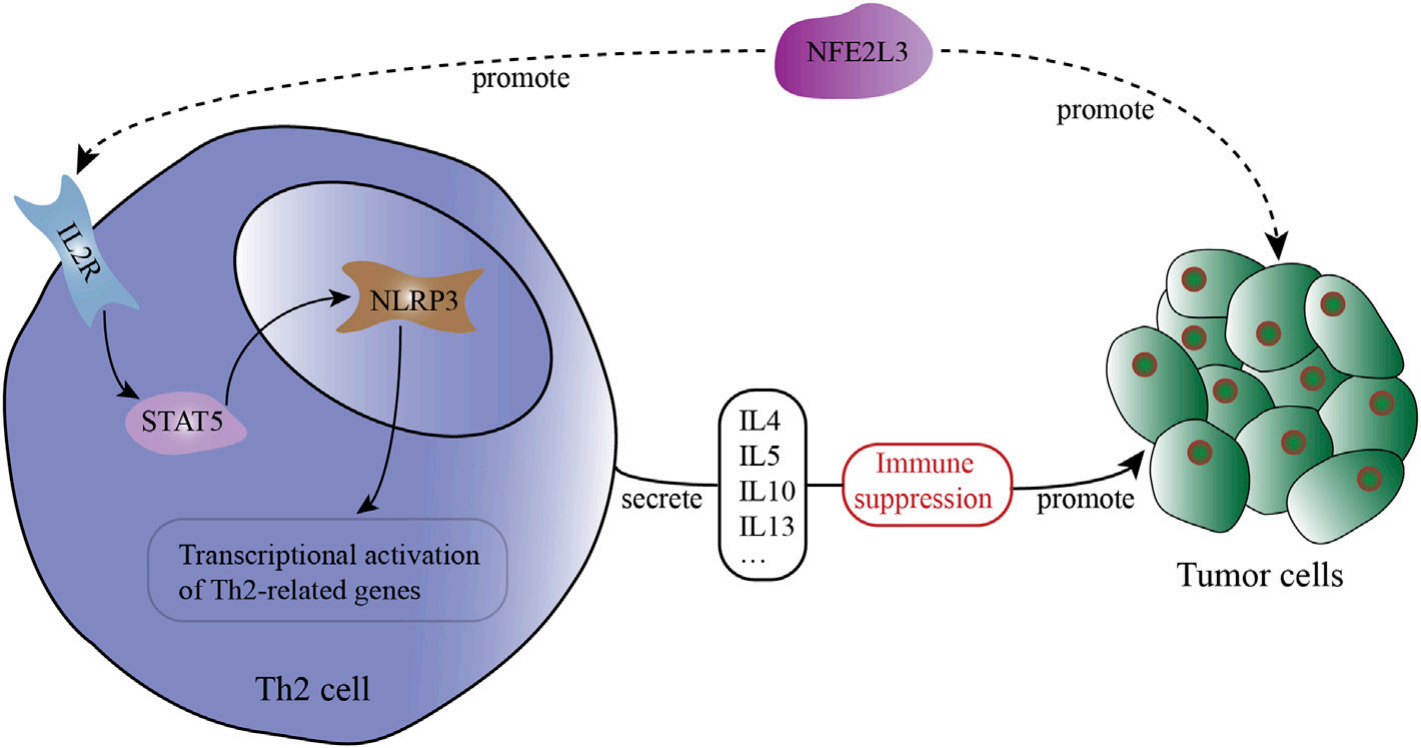
The potential interactions across NFE2L3, IL-2R, STAT5, NLRP3, Th2 cell and tumor cells in the tumor microenvironment (TME).

As shown in Table 2, in MESO, NFE2L3 has a strong correlation with IL-2RA (*Cor* = 0.295), IL-2RB (*Cor* = 0.303) and IL-2RG (*Cor* = 0.228), which constitute the high-affinity IL2 receptor (IL-2R) (Wang et al., 2005). STAT5A and STAT5B are part of JAK/STAT signaling pathway, they can mediate transcriptional signals by forming homodimers or heterodimers (Maurer et al., 2019). In MESO, NFE2L3 and STAT5B show a strong correlation (Table 2, *Cor* = 0.35), but the correlation between NFE2L3 and STAT5A is not statistically significant. And there is also a strong correlation between NFE2L3 and NLRP3 (Table 2, *Cor* = 0.254). The potential interactions across NFE2L3, IL-2R, STAT5, NLRP3, Th2 cell and tumor cells are shown in Figure 6.

**TABLE 2:** Correlation analyses between NFE2L3 and IL- 2R/STAT5/NLRP3 related genes in TIMER, which were adjusted by tumor purity. MESO, mesothelioma; BRCA (Her2), Her2 positive breast invasive carcinoma; DLBC, diffuse large B-cell lymphoma; ESCA, esophageal carcinoma; HNSC-HPVpos, HPV positive head and neck cancer; KIRC, kidney renal clear cell carcinoma; KIRP, kidney renal papillary cell carcinoma; LGG, low grade glioma; LIHC, liver hepatocellular carcinoma; LUSC, lung squamous cell carcinoma; PRAD, prostate adenocarcinoma; SARC, sarcoma; SKCM, skin cutaneous melanoma; THCA, thyroid carcinoma; Cor, R value of Spearman’s correlation.

		NFE2L3					
		IL2RA	IL2RB	IL2RG	STAT5A	STAT5B	NLRP3
MESO	Cor	0.295	0.303	0.228	0.085	0.35	0.254
	*p*-value	6.13E-03^*^	4.75E-03^*^	3.58E-03^*^	4.40E-01	1.03E-03^*^	1.89E-02^*^
BRCA (Her2)	Cor	0.443	0.491	0.459	0.49	0.315	0.417
	*p*-value	5.04E-04^*^	8.94E-05^*^	2.86E-04^*^	9.51E-05^*^	1.59E-02^*^	1.12E-03^*^
DLBC	Cor	0.418	0.719	0.311	0.098	0.587	0.638
	*p*-value	6.60E-03^*^	1.22E-07^*^	4.80E-02^*^	5.42E-01	5.57E-05^*^	7.29E-06^*^
ESCA	Cor	0.249	0.228	0.435	0.377	0.289	0.251
	*p*-value	7.63E-04^*^	2.10E-03^*^	1.06E-09^*^	1.89E-07^*^	8.41E-05^*^	6.64E-04^*^
HNSC (HPVpos)	Cor	0.456	0.612	0.671	0.566	0.227	0.275
	*p*-value	7.24E-06^*^	1.83E-10^*^	6.12E-13^*^	7.39E-09^*^	3.24E-02^*^	9.11E-03^*^
KIRC	Cor	0.317	0.423	0.355	0.359	-0.006	0.357
	*p*-value	3.15E-12^*^	1.74E-21^*^	4.05E-15^*^	1.73E-15^*^	9.03E-01	2.89E-15^*^
KIRP	Cor	0.33	0.344	0.304	0.396	0.434	0.391
	*p*-value	5.65E-08^*^	1.34E-08^*^	6.16E-07^*^	4.12E-11^*^	3.03E-13	7.41E-11^*^
LGG	Cor	0.169	0.294	0.324	0.427	0.241	0.28
	*p*-value	2.10E-04^*^	5.54E-11^*^	3.77E-13^*^	1.48E-22^*^	9.89E-08	4.40E-10^*^
LIHC	Cor	0.496	0.532	0.573	0.45	0.324	0.551
	*p*-value	7.62E-23^*^	1.49E-26^*^	1.61E-31^*^	1.32E-18^*^	7.51E-10^*^	9.91E-29^*^
LUSC	Cor	0.229	0.27	0.322	0.328	0.168	0.158
	*p*-value	4.38E-07^*^	2.02E-09^*^	5.81E-13^*^	1.99E-13^*^	2.31E-04^*^	5.36E-04^*^
PRAD	Cor	0.297	0.349	0.344	0.166	0.278	0.301
	*p*-value	6.04E-10^*^	2.30E-13^*^	5.43E-13^*^	7.02E-04^*^	7.89E-09^*^	3.90E-10^*^
SARC	Cor	0.249	0.395	0.401	0.207	0.062	0.432
	*p*-value	8.42E-25^*^	1.55E-10^*^	8.06E-11^*^	1.12E-03^*^	3.31E-01	1.64E-12^*^
SKCM	Cor	0.43	0.225	0.187	0.003	0.304	0.421
	*p*-value	5.84E-22^*^	1.16E-06^*^	5.56E-05^*^	9.55E-01	3.23E-11^*^	4.78E-21^*^
THCA	Cor	0.632	0.543	0.54	0.312	0.03	0.398
	*p*-value	9.24E-56^*^	8.45E-39^*^	2.83E-38^*^	1.86E-12^*^	5.12E-01	5.66E-20^*^

In addition to MESO, NFE2L3 also shows powerful correlations with IL-2RA, IL-2RB and IL-2RG in many other tumors, including BRCA-Her2, DLBC, ESCA, HNSC-HPVpos, KIRC, KIRP, LGG, LIHC, LUSC, PRAD, SARC, SKCM and THCA (Table 2). And among the above cancers, NFE2L3 shows a strong correlation with STAT5A or STAT5B as well (Table 2). Among the cancers analyzed, the correlations between NFE2L3 and NLRP3 are also statistically significant, among which the correlation is relatively low in LUSC (Table 2, *Cor* = 0.158), and the correlation is highest in DLBC (Table 2, *Cor* = 0.638).

## Discussion

As a very aggressive malignant tumor, malignant pleural mesothelioma (MPM) is believed to be closely related to asbestos exposure, BRCA1-associated protein 1 (BAP1) mutation and ionizing radiation to chest (Carbone et al., 2019). And a variety of somatic mutations including BAP1, TP53, NF2 and LATS1/2, are closely related to the occurrence and development of MPM (Bueno et al., 2016; Hmeljak et al., 2018; Yang et al., 2020a; Yang et al., 2020b). Due to the low incidence of MPM and the difficulty in diagnosis, there is still no unified and effective model for its treatment. Surgery used to be the only treatment for MPM, but the indications, extent of surgical resection and survival benefits of surgery are still controversial (Schipper et al., 2008; Cao et al., 2014). Beginning in 2004, the US Food and Drug Administration (FDA) approved the combination of cisplatin and pemetrexed as the first-line regimen for treatment of mesothelioma (Vogelzang et al., 2003). Although it has dramatically improved the survival of patients, the median survival period of patients who received combined chemotherapy after surgery still hovered between 17 and 25 months (Tsao et al., 2018).

Of note, with the advent of the age of immunotherapy, a variety of immune checkpoint inhibitors (ICIs) have brought a new dawn to the treatment of MPM. The DREAM study (Durvalumab with First-Line Chemotherapy in Mesothelioma) investigated the combination of PD-L1 inhibitor durvalumab and first-line chemotherapy (cisplatin and pemetrexed), and has brought survival benefits to patients (Nowak et al., 2020). Then a subsequent international, randomized, phase 3 study (CheckMate743) investigated Nivolumab in combination with Ipilimumab versus Pemetrexed with Cisplatin or Carboplatin as first line treatment in unresectable MPM, and identified that dual immune checkpoint inhibitors could bring long-term survival for these patients regardless of histological type (Baas et al., 2021).

Therefore, a better understanding of the tumor immune microenvironment and the construction of a more precise immune regulatory network will bring more individualized immunotherapy and survival benefits to patients. However, the tumor immune microenvironment is shaped by tumor cells and immunocytes together, and is in dynamic change (Schreiber et al., 2021). It is well known that CD4^+^ T cell populations are abundant in this environment, including pro-tumor CD4^+^ regulatory T cells (Tregs) and anti-tumor Th1 cells, but the role of Th2 cells is not clear so far (Facciabene et al., 2012; Tay et al., 2021).

In our study, the patients with high Th2 cell infiltration levels suffer a poor prognosis, which is consistent with some previous studies that Th2 cells are associated with tumor progression and poor prognosis in many cancers such as pancreatic cancer, breast cancer and melanoma (Nevala et al., 2009; De Monte et al., 2011; Zhang et al., 2015). The promotion effect of Th2 cells on tumors is probably due to the cytokines secreted by them, including IL-4, IL-5, IL-10 and IL-13 (Lee, 2014; Mollazadeh et al., 2019). IL-4 and IL-13 are highly similar in structure and function (Shi et al., 2021). After binding to their receptors, they can promote the proliferation, adhesion and metastasis of tumor cells, and may become potential targets for tumor treatment (Suzuki et al., 2015; Ghilardi et al., 2020). IL-5 can create a tumor-promoting immune microenvironment locally by recruiting eosinophils, thereby promoting the metastasis of tumor cells (Zaynagetdinov et al., 2015; Reichman et al., 2016). IL-10 can create an immunosuppressive tumor microenvironment through multiple pathways including NF-κB, and promote the transformation of cancer stemness (Yang et al., 2019; Saraiva et al., 2020).

Through further analysis, both the mRNA expression level and DNA methylation level of NFE2L3 was found to be highly correlated with the infiltration level of Th2 cells. NFE2L3 is a family member of the Cap’n’collar (CNC) transcription factors, and this family also include NFE2L1, NFE2L2, NF-E2, Bach1 and Bach2 (Ren et al., 2020). Among them, the family member that is widely investigated is NFE2L2, which has been confirmed as a driver gene of malignant tumor (DeNicola et al., 2011). As a homolog of NFE2L2, NFE2L3 has been proven to be related to multiple phenotypes of malignant tumors as well, including proliferation and epithelial-mesenchymal transition (EMT) (Bury et al., 2019; Ren et al., 2020). In our study, NFE2L3 was detected to be highly expressed in MPM tumor tissues, and the higher expression level is associated with poor prognosis. And the hypermethylation of multiple sites of NFE2L3 was also associated with better prognosis of patients with MPM. In multiple previous studies, somatic mutations of NFE2L2, the homolog of NFE2L3, was detected in plasma cell-free DNA (cfDNA) in hepatocellular carcinoma (HCC) and lung squamous cell carcinoma (LUSC), and was regarded as a non-invasive biomarker for tumor risk prediction and overall survival (Jeong et al., 2017; Jiao et al., 2021). Therefore, through next-generation sequencing (NGS) of tumor tissues and detection of cfDNA in peripheral blood, NFE2L3 may serve as a potential marker for the diagnosis and prognosis prediction of patients with MPM.

Then, we explored the underlying mechanism between NFE2L3 expression and Th2 cell differentiation. Th2 cell differentiation can be activated and modulated by a variety of regulators, including IL-4/STAT6 signaling pathway, IL-2/STAT5 signaling pathway and transcriptional regulator NLRP3, of which NLRP3 expression is triggered via IL-2/STAT5 signaling pathway (Lee, 2014; Bruchard et al., 2015; Jones et al., 2020). NLRP3 inflammasome is a kind of cytoplasmic protein complex, which has been proven that it can recruit myeloid-derived suppressor cells (MDSCs) and tumor-associated macrophages (TAMs) to promote tumor progression and metastasis (Moossavi et al., 2018; Hamarsheh and Zeiser, 2020). In the process of Th2 cells differentiation, NLRP3 was found to be localized in the nucleus and act as a transcription factor for Th2 cells (Bruchard et al., 2015).

In our study, the expression of NFE2L3 shows strong positive correlations with the expression of IL-2 receptor-related genes, STAT5 related genes and NLRP3. These correlations can be observed in multiple cancers, which are even more significant. Taken together, we speculated that NFE2L3, a novel biomarker in malignant pleural mesothelioma, can promote Th2 cell differentiation *via* IL-2/STAT5/NLRP3 signaling pathway in mesothelioma and many other cancers.

We acknowledge that there are several limitations in this study. In this article, our analysis is based only on transcriptome and DNA methylation data from TCGA and GEO without biological validation. Therefore, complete biological experiments are urgently needed to verify our conclusions in the future.

## Conclusion

In our study, with the help of transcriptome data from multiple databases and a variety of bioinformatics analysis methods, we found that Th2 cell is a poor prognostic factor for patients with MPM. Through further screening, we found that NFE2L3 was highly expressed in tumor tissues of patients with MPM and both its mRNA expression level and DNA methylation level was highly correlated with the infiltration level of Th2 cells. Moreover, the correlation analysis in multiple cancers indicated that NFE2L3 was strongly correlated with the expression level of IL-2RA, IL-2RB, IL-2RG, STAT5A, STAT5B and NLRP3, which constitute the IL-2/STAT5/NLRP3 signaling pathway. Therefore, we hypothesize that NFE2L3, a novel biomarker in malignant pleural mesothelioma, may promote the differentiation of Th2 cells through the IL-2/STAT5/NLRP3 signaling pathway in multiple cancers.

## Data Availability

The original contributions presented in the study are included in the article Supplementary Material, further inquiries can be directed to the corresponding author.
