# Supplementary figures and images for: NFE2L3 as a Novel Biomarker Associated With IL-2/STAT5/NLRP3 Signaling Pathway in Malignant Pleural Mesothelioma and Other Cancers
Source: Front Genet. 2022 May 18;13:805256. doi: 10.3389/fgene.2022.805256 (PMC9158472; doi:10.3389/fgene.2022.805256)

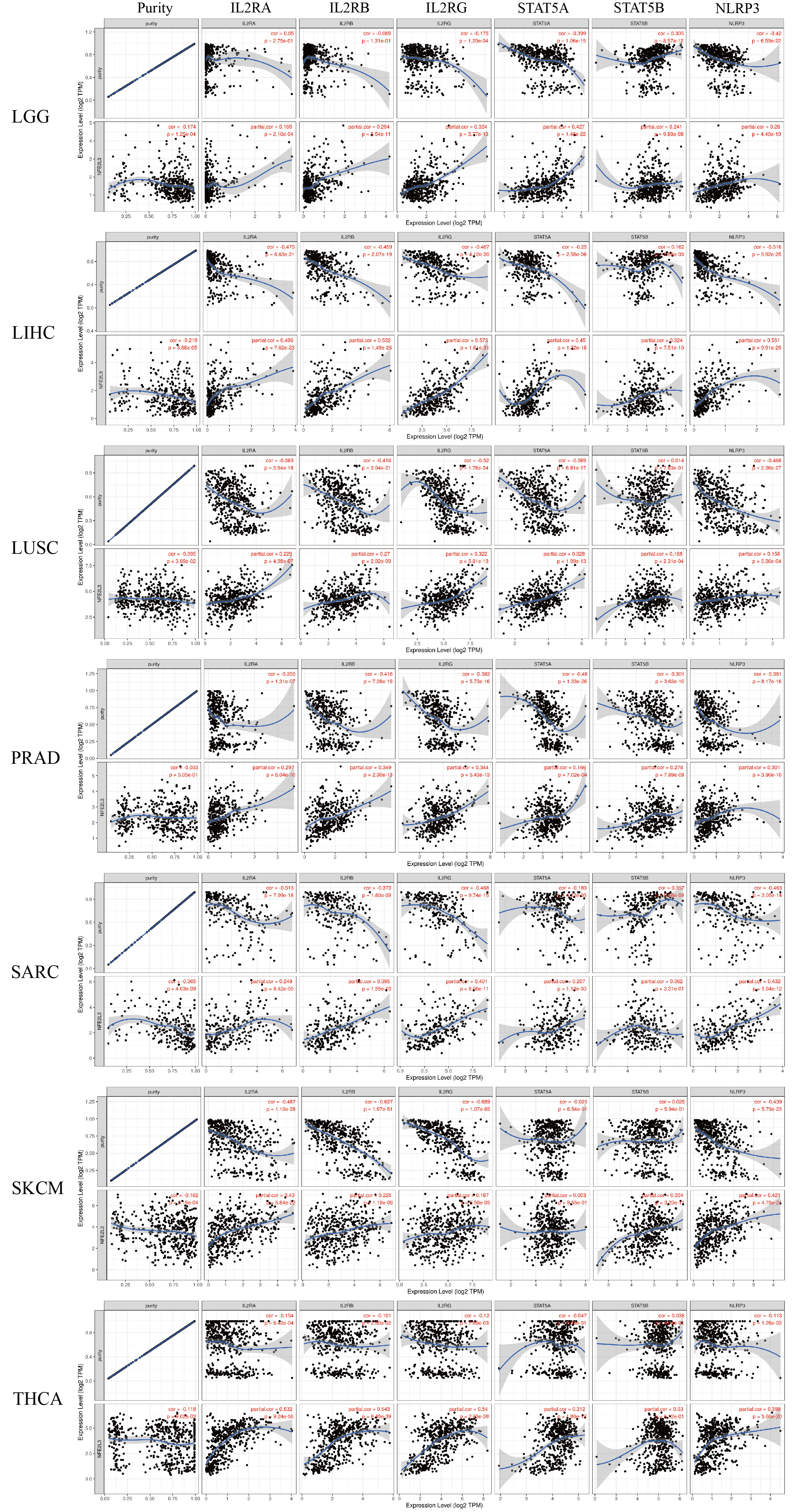

Supplement: Supplementary file 1 [file Image3.JPEG]

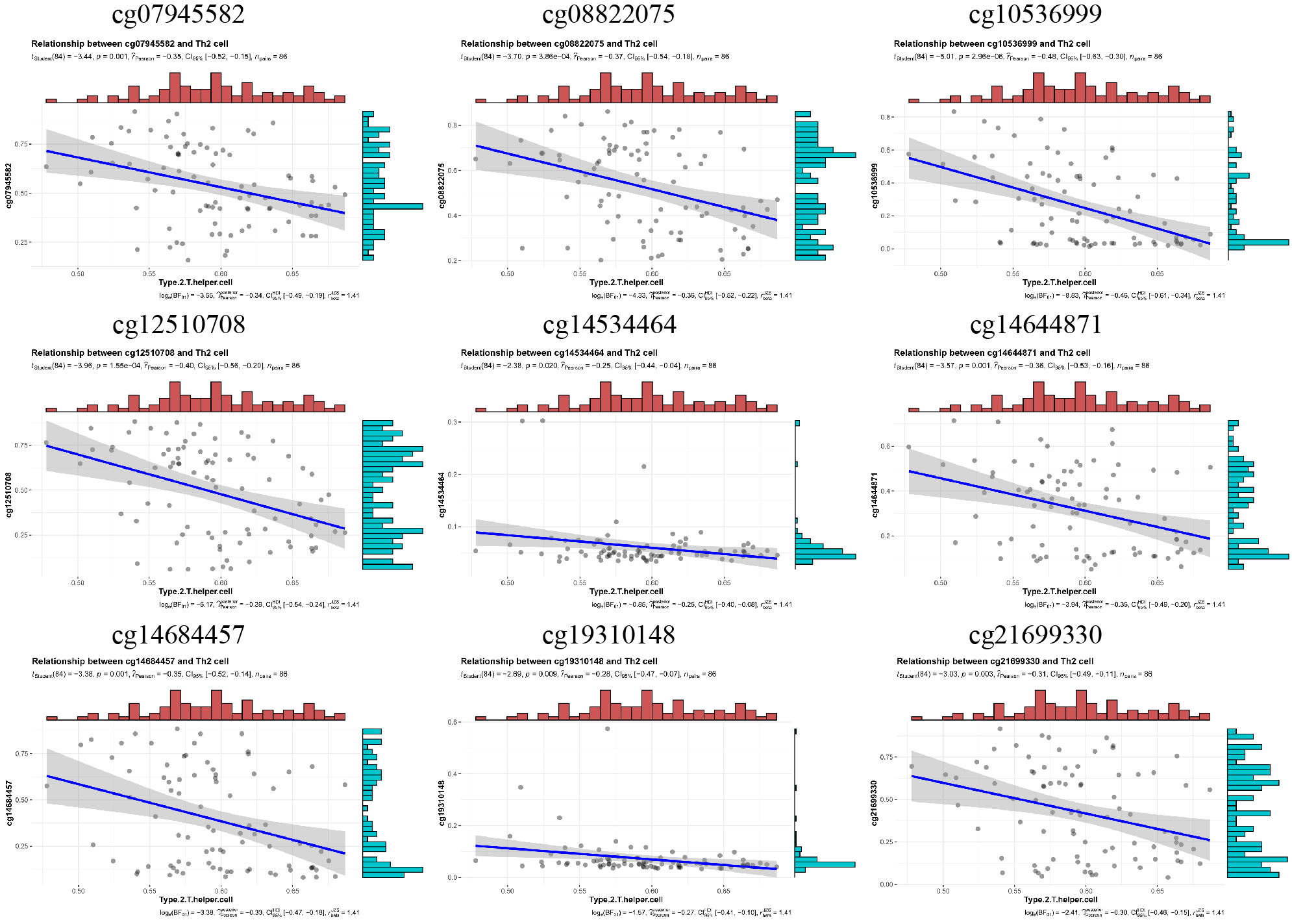

Supplement: Supplementary file 2 [file Image1.JPEG]

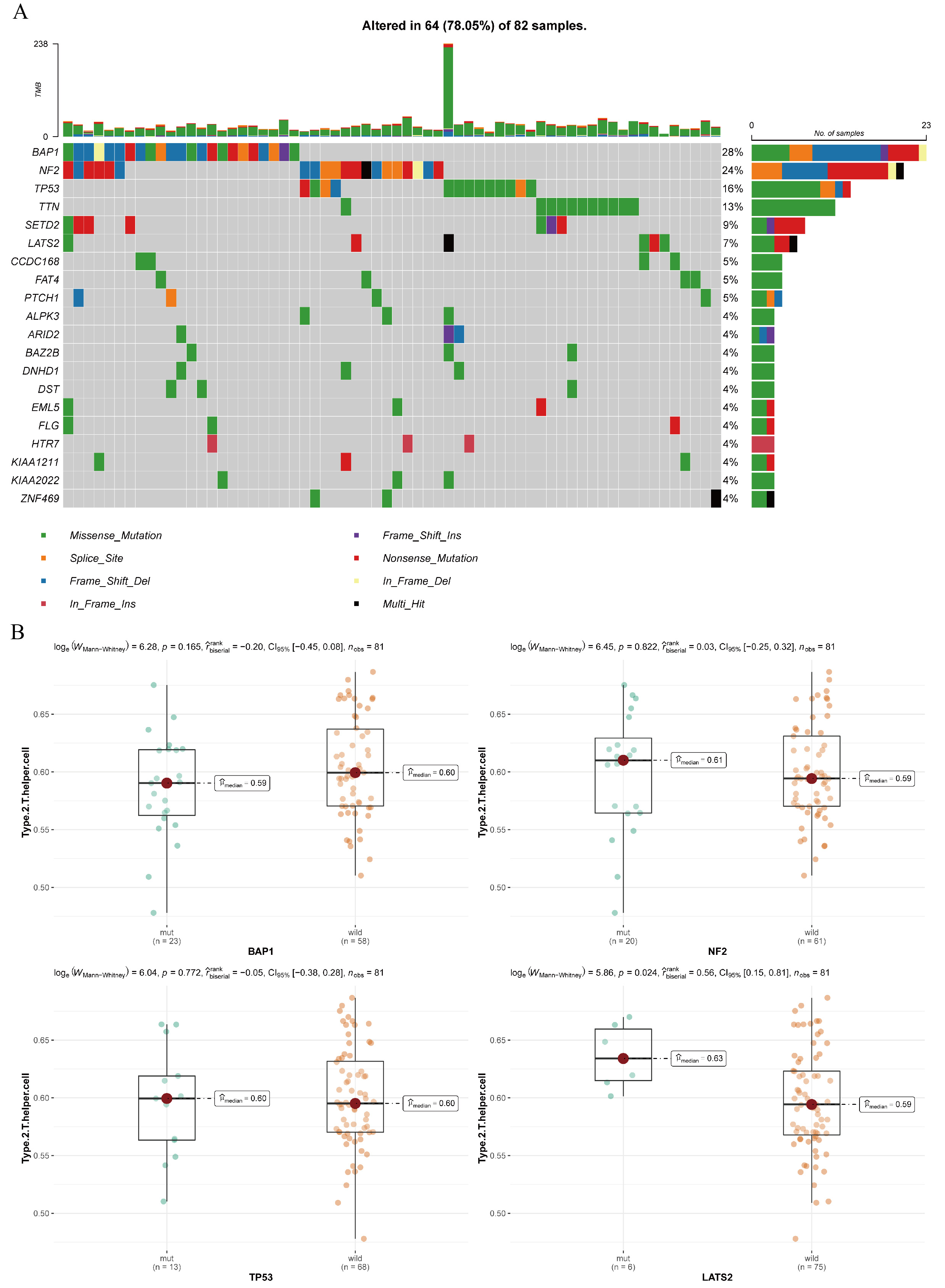

Supplement: Supplementary file 3 [file Image4.JPEG]

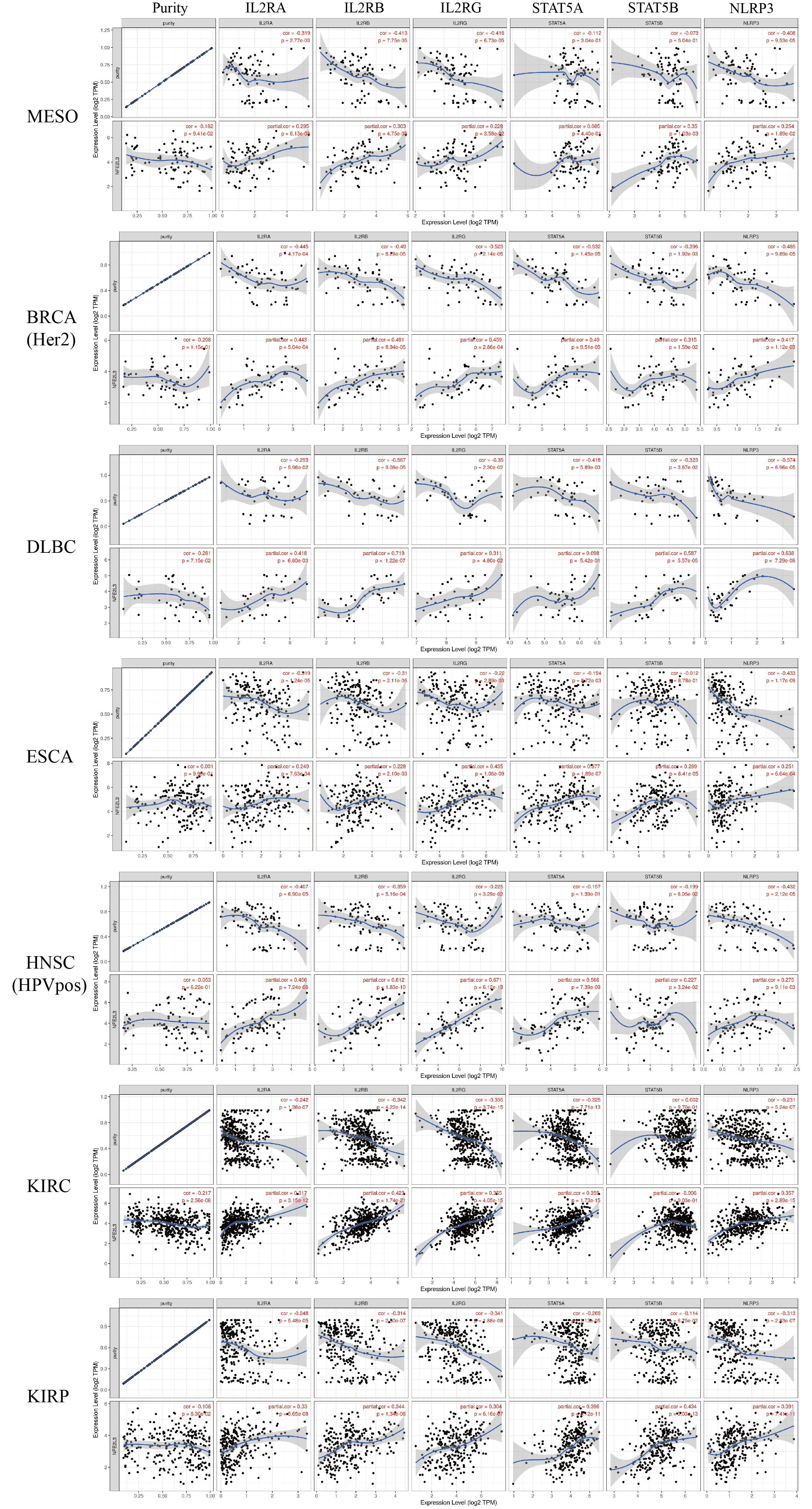

Supplement: Supplementary file 4 [file Image2.JPEG]

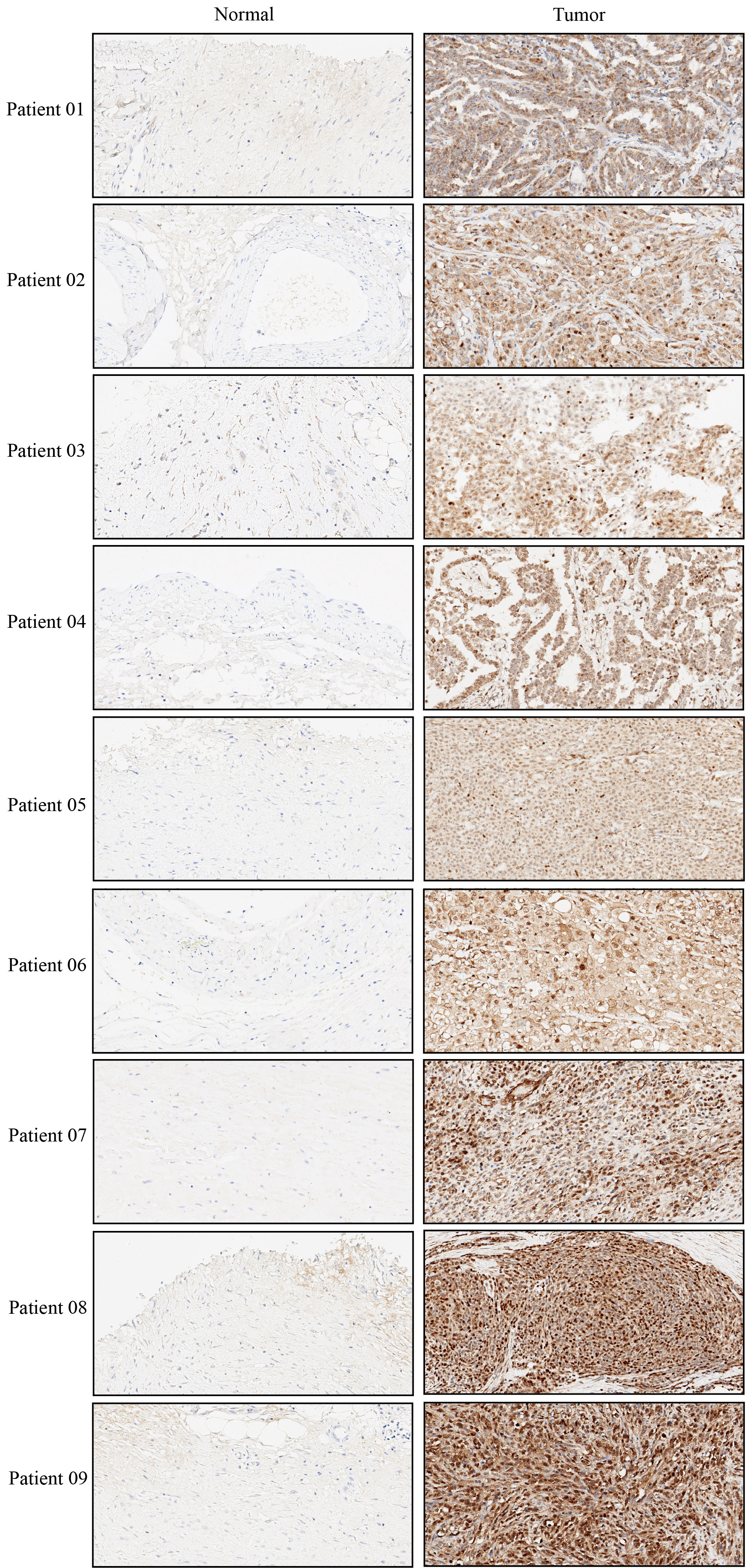

Supplement: Supplementary file 5 [file Image5.JPEG]
