# Supplementary material for: NFE2L3 as a Novel Biomarker Associated With IL-2/STAT5/NLRP3 Signaling Pathway in Malignant Pleural Mesothelioma and Other Cancers
Source: Front Genet. 2022 May 18;13:805256. doi: 10.3389/fgene.2022.805256 (PMC9158472; doi:10.3389/fgene.2022.805256)
Supplement: Supplementary file 6 [file Table2.DOCX]

**Supplementary-Figure-1.** Correlations between methylation levels of NFE2L3 DNA methylation sites and Th2 cell infiltration levels.

**Supplementary-Figure-2.** Correlation analyses between NFE2L3 and IL-2R/STAT5/NLRP3 related genes in TIMER, which were adjusted by tumor purity. MESO, mesothelioma; BRCA (Her2), Her2 positive breast invasive carcinoma; DLBC, diffuse large B-cell lymphoma; ESCA, esophageal carcinoma; HNSC-HPVpos, HPV positive head and neck cancer; KIRC, kidney renal clear cell carcinoma; KIRP, kidney renal papillary cell carcinoma; Cor, R value of Spearman’s correlation.

**Supplementary-Figure-3.** Correlation analyses between NFE2L3 and IL-2R/STAT5/NLRP3 related genes in TIMER, which were adjusted by tumor purity. LGG, low grade glioma; LIHC, liver hepatocellular carcinoma; LUSC, lung squamous cell carcinoma; PRAD, prostate adenocarcinoma; SARC, sarcoma; SKCM, skin cutaneous melanoma; THCA, thyroid carcinoma; Cor, R value of Spearman’s correlation.

**Supplementary-Figure-4.** (A) Landscape of the mutation profiles in mesothelioma samples from TCGA. (B) Relationship between NFE2L3 expression level and common mutations in mesothelioma.

**Supplementary-Figure-5.** The results of immunohistochemical staining in 9 patients with MPM.

**mmc3.** Metagenes that were used in immune infiltration scoring in this article, which were derived from the supplementary material of the published literature (PMID: 28052254).
